# Microbial biocontrol agents and the rhizosphere microbiome: integrating ecological function and climate resilience in sustainable agriculture

**DOI:** 10.3389/fmicb.2026.1771649

**Published:** 2026-02-26

**Authors:** Mudassir Iqbal

**Affiliations:** Department of Plant Protection Biology, Swedish University of Agricultural Sciences, Lomma, Sweden

**Keywords:** biocontrol agents, integrated pest management, microbial consortia, plant–microbe interactions, rhizosphere microbiome, soil health

## Abstract

The growing challenges of food insecurity, soil degradation, and climate-induced stresses are exposing the limitations of chemically intensive crop protection systems. In this context, the rhizosphere microbiome, comprising complex microbial networks that regulate plant growth, nutrient acquisition, and immune responses, has emerged as a promising focus for more sustainable agricultural practices. Microbial biocontrol agents (BCAs) are increasingly recognized not only for their pathogen-suppressive properties but also for their potential to modulate rhizosphere microbial communities and contribute to plant tolerance to abiotic stressors. This review synthesizes recent advances in understanding the ecological and mechanistic interplay between BCAs and the rhizosphere microbiome, highlighting how microbial inoculants can influence community assembly, functional processes, and microbiome resilience under biotic and abiotic stress conditions. Drawing on molecular and ecological evidence, the synthesis integrates current knowledge of BCA-mediated regulation of plant defense signalling, nutrient cycling, and stress-associated responses. Key knowledge gaps related to inoculant persistence, ecological compatibility, and microbiome-level trade-offs that limit field-scale effectiveness are also identified. To address these challenges, a microbiome-informed conceptual framework is proposed, emphasizing precision-designed synthetic microbial communities (SynComs), trait-based screening, host–microbiome co-optimization, and integration of BCAs into resilient Integrated Pest Management (IPM) strategies. In summary, this review provides a systems-level perspective on how rhizosphere microbiome dynamics can be leveraged to support sustainable climate-smart crop production.

## 1 Introduction

Agricultural systems worldwide are facing challenges in ensuring food security for a rapidly growing population, which is expected to exceed 9.7 billion by 2050 (Suman et al., 2022; United Nations, 2019). To meet this demand, an estimated 70% increase in food production will be required (Poveda, 2021). However, agriculture must rely on limited cultivable land that is increasingly threatened by climate change, land degradation associated with declining soil function, and unsustainable farming practices. Climate-induced abiotic stressors such as drought, erratic rainfall, rising temperatures, and soil salinization are already disrupting plant physiological processes and reducing yields (Park et al., 2023; Zandalinas et al., 2022). At the same time, shifting environmental conditions are reshaping plant–pathogen interactions, accelerating the spread, persistence, and virulence of destructive plant pathogens including bacteria, fungi, oomycetes, viruses, and nematodes (Singh et al., 2023). Collectively, these biotic stressors contribute to an estimated $220 billion in annual crop losses (Ristaino et al., 2021; Rohr et al., 2019; Van Dijk et al., 2021) that is further exacerbated by post-harvest deterioration (Srivastava, 2019), posing a direct threat to global food security.

While current management relies heavily on synthetic chemical inputs, these conventional approaches are increasingly proving inadequate under escalating biotic and abiotic pressures. These limitations are not only due to declining efficacy and the rise of resistant pathogen strains (Fisher et al., 2018; Lucas et al., 2015), but also to their unintended consequences for environmental health (Pathak et al., 2022), biodiversity (Sánchez-Bayo and Wyckhuys, 2019), and human well-being (Asghar et al., 2016). The overreliance on synthetic pesticides and fertilizers has been linked to soil microbiome disruption, contamination of water bodies, bioaccumulation in food chains, and negative impacts on pollinators and beneficial organisms (Devi et al., 2018; Goulson et al., 2015). Additionally, these practices raise serious concerns about food safety and long-term sustainability, necessitating the urgent development of ecologically sound alternatives.

As a result, growing attention has turned to the rhizosphere microbiome, a dense and dynamic microbial consortium at the soil–root interface that functions as a fundamental determinant of plant health, stress tolerance, and disease resistance (Philippot et al., 2013). These microbial communities reinforce a wide array of physiological and ecological functions, including nutrient cycling, phytohormone signalling, modulation of root architecture, and immune priming, while simultaneously acting as a biological barrier against soilborne pathogens (Busby et al., 2017; Park et al., 2023). The rhizosphere microbiome’s sensitivity to environmental cues and high functional plasticity reflect its function as a buffer against climatic stressors and mark it out as a promising target for agroecological innovation (Trivedi et al., 2022). Microbiome manipulation and targeted microbiome modulation are thus increasingly recognized as pivotal strategies for advancing climate-smart and resilient agricultural systems while avoiding the limitations of chemical-intensive practices (Han and Yoshikuni, 2022; Kaur et al., 2025). However, deeper knowledge is required to enable predictable, ecologically compatible, and field-relevant intentional microbiome manipulation.

While the rhizosphere microbiome’s central role in plant health and stress resilience has been recognized, the availability of practical and scalable tools for its manipulation is limited. Microbial biological control agents (BCAs) may represent one such tool. Several studies have examined the ability of microbial BCAs to suppress plant pathogens (Iqbal et al., 2021, 2023; Thangaraj et al., 2025) and the native microbiome’s impact on crop performance (Busby et al., 2017; Chandel et al., 2019; Philippot et al., 2013). Conversely, few have explored how BCAs interact with, restructure, or integrate into rhizosphere microbial communities to enhance plant resilience under combined biotic and abiotic stresses. Understanding of these issue is particularly important in the context of climate-resilient agriculture and integrated pest management (IPM) systems, where sustainability depends not only on effectiveness but also on ecological compatibility. In this context, ecological compatibility refers to the capacity of introduced BCAs to establish, persist, and function within native rhizosphere communities while maintaining beneficial microbial interactions and essential ecosystem processes (Muhammad et al., 2024; Stenberg, 2017; Whipps, 2001). Despite its importance, the persistence, colonization, and functional influence of BCAs within native microbiomes remain poorly explored across crop systems and environmental gradients.

**Figure 1:**
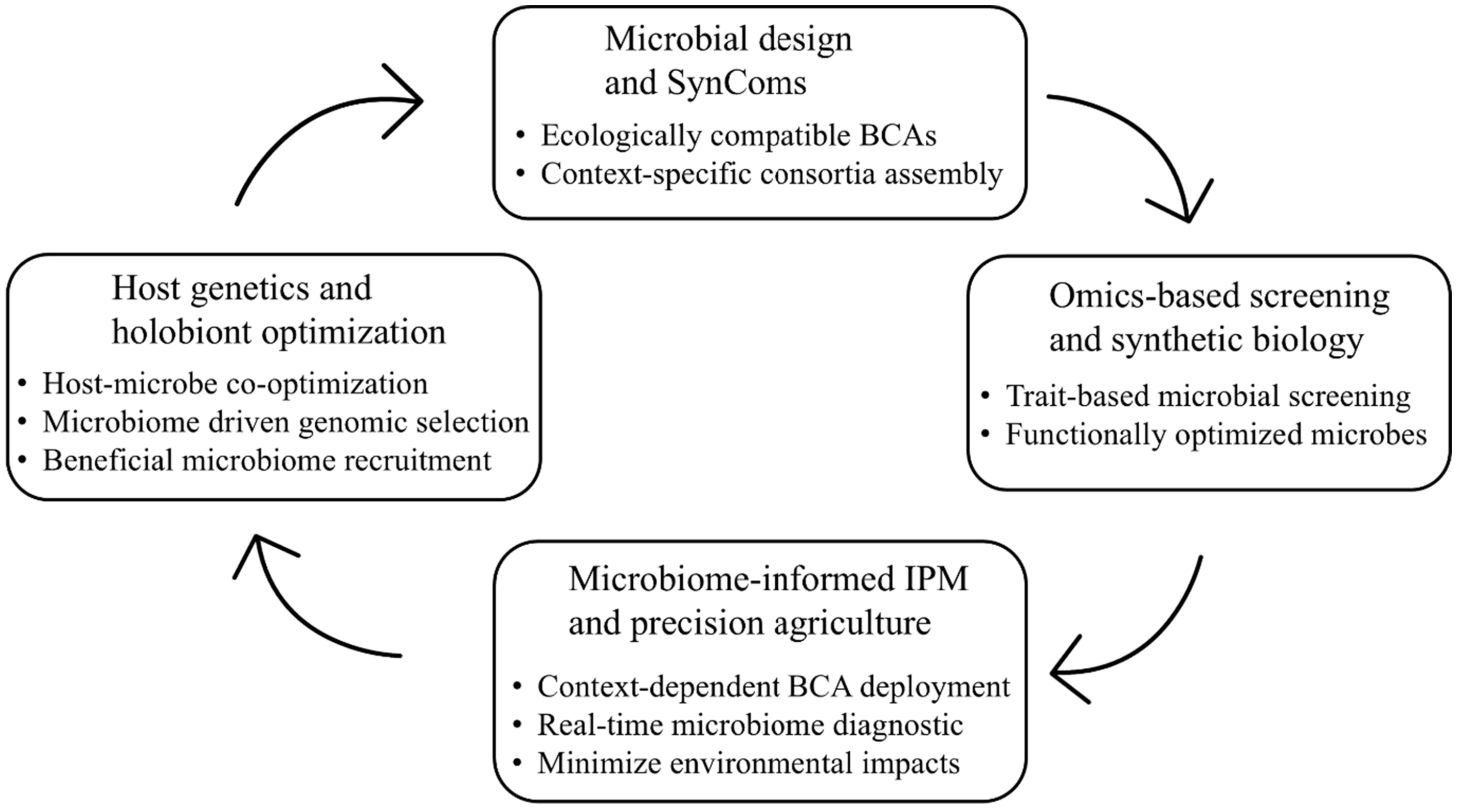
Conceptual framework for microbiome-informed biological control in climate-smart agriculture. The framework illustrates an iterative, systems-level approach integrating consortium-level microbial design, trait-based screening and optimization, microbiome-informed integrated pest management, and host–microbiome co-optimization. By explicitly incorporating microbial functional traits, ecological compatibility with resident microbiomes, and validation across environmental gradients, the framework extends conventional IPM strategies toward context-dependent, resilient disease management under climate stress.

In this review, I synthesize current understanding of the ecological interactions between microbial BCAs and the rhizosphere microbiome. Specifically, I discuss the mechanisms through which BCAs influence microbial community assembly and function, highlight case studies illustrating crop-specific and context-dependent outcomes, and assess the implications for sustainable disease management and climate adaptation. Finally, I propose a conceptual framework for microbiome-informed biocontrol strategies (Figure 1) that align with the principles of IPM and climate-smart agriculture, integrating near-term deployable approaches with longer-term innovation pathways, and advancing a systems-level approach to enhancing crop resilience and sustainable productivity.

## 2 The rhizosphere microbiome: composition, function, and environmental sensitivity

### 2.1 Rhizosphere microbiome

The rhizosphere microbiome is structured by complex, dynamic interactions between plant genotype, soil characteristics, and environmental conditions. It plays an important role in determining plant health, nutrient cycling, and overall agroecosystem functionality (Busby et al., 2017; Massart et al., 2015). While soil is the primary reservoir of microbial inocula, its physicochemical properties such as pH, texture, organic matter content, and water-holding capacity strongly influence the baseline microbial community available for root colonization (Berg and Smalla, 2009; Berg et al., 2013). In the rhizosphere, this soil-derived pool is further filtered by plant-specific traits including root architecture, exudate composition, and immune signalling pathways. Experimental evidence demonstrates that even within identical soil types, different plant genotypes can assemble distinct microbial consortia, highlighting the host’s regulatory role in microbiome assembly (Ikeda et al., 2011; Park et al., 2023).

In addition to inherent plant–soil interactions, anthropogenic factors such as fertilization regimes, pesticides, and cropping patterns significantly shape rhizosphere microbiome structure and function. Fertilizers, particularly those applied in excess, have been linked to reduced microbial diversity and functional redundancy, potentially impairing key processes like nutrient cycling and pathogen suppression (Jacobsen and Hjelmsø, 2014). Conversely, practices such as crop rotation and organic amendments tend to promote microbial resilience, enhance beneficial taxa, and suppress soilborne pathogens (Hilton et al., 2013). These findings challenge the concept of the rhizosphere as a passive microbial habitat and instead highlight its dynamic responsiveness to agronomic management. Within the broader framework of climate-smart agriculture, the rhizosphere represents not only a site of biological activity but also a manipulable interface through which plant resilience, productivity, and sustainability can be enhanced.

### 2.2 Composition and key microbial groups

The rhizosphere microbiome is a phylogenetically diverse and functionally heterogeneous assemblage of microorganisms including bacteria, fungi, archaea, protists, and viruses that collectively shape plant–soil interactions (Chukwuneme and Babalola, 2025). These groups have complementary ecological functions that influence nutrient cycling, plant defense, and stress responses.

Bacteria are the most abundant and metabolically versatile members of the rhizosphere microbiome. Dominant phyla such as Proteobacteria, Actinobacteria, Firmicutes, and Bacteroidetes contribute to key functions including nutrient cycling, siderophore production, phytohormone modulation, and pathogen suppression (Fierer, 2017; Kour et al., 2023). Bacterial community composition is highly dynamic and shaped by host genotype, soil properties, and environmental conditions, with stress factors often favoring specific functional groups, such as Proteobacteria under nitrogen limitation and Actinobacteria under drought (Negre Rodríguez et al., 2025; Sun et al., 2024). Within this context, keystone members of the rhizosphere microbiome include both functionally distinct groups and taxonomically defined but functionally versatile genera. For example, diazotrophs act as keystone functional groups through their contribution to biological nitrogen fixation (Imran et al., 2021), whereas *Pseudomonas* spp. represent keystone genera that influence community structure and plant performance via pathogen suppression, siderophore production, and induced systemic resistance (Mazzola, 2002; Trivedi et al., 2022).

Fungi are another major functional component of the rhizosphere microbiome. Filamentous fungi from Ascomycota and Basidiomycota contribute to organic matter decomposition, phosphorus solubilization, and pathogen suppression through mycoparasitism and secondary metabolite production (Chapelle et al., 2016; Rigobelo, 2025). Arbuscular mycorrhizal fungi (AMF; Glomeromycota) form symbiotic associations with plant roots, enhancing nutrient acquisition, water-use efficiency, and tolerance of abiotic stresses such as drought and salinity while also influencing soil structure and host immune responses (Nie et al., 2024; Wahab et al., 2023). Fungal community composition is strongly shaped by plant genotype, soil pH, and nutrient availability.

Archaea, although generally less abundant than bacteria and fungi, play important roles in rhizosphere biogeochemical cycling, particularly in ammonia oxidation and anaerobic methanogenesis (Offre et al., 2013; Prosser, 2012). These processes are especially relevant in nutrient-poor, saline, or anoxic soils, where archaeal activity complements bacterial metabolism. Protists and viruses are key regulators of rhizosphere microbial dynamics. Protists influence microbial turnover through selective bacterivory, thereby affecting nutrient mineralization and community succession (Liao et al., 2024). Bacteriophages shape bacterial populations through lytic and lysogenic interactions that regulate population dynamics and mediate horizontal gene transfer, thereby contributing to microbial adaptation and functional stability under changing environmental conditions (Chevallereau et al., 2022). Collectively, interactions among these microbial guilds underpin the ecological functioning and resilience of the rhizosphere. Table 1 summarizes the major rhizosphere microbial groups, showing representative taxa from each and their dominant functional roles.

**Table 1:** Major microbial groups inhabiting the rhizosphere, representative taxa, and their dominant functional roles relevant to plant health, disease regulation, and stress resilience.

Microbial groups	Representative taxa (examples)	Dominant functional roles	Relevance to plant health and stress	References
Beneficial bacteria	*Pseudomonas, Bacillus, Azospirillum*	Nutrient solubilization, phytohormone modulation, ISR induction, antibiosis	Enhanced nutrient uptake, disease suppression, drought tolerance	Fierer (2017), Mazzola (2002)
Beneficial fungi	*Trichoderma*, AMF (Glomeromycota)	Mycoparasitism, nutrient acquisition, ISR, improved water uptake	Pathogen suppression, abiotic stress tolerance	Chapelle et al. (2016), Nie et al. (2024)
Plant pathogens	*Fusarium, Ralstonia, Rhizoctonia*	Host colonization, microbiome disruption	Disease development, microbiome dysbiosis	Berendsen et al. (2012), Deng et al. (2021), Su et al. (2020)
Archaea	Ammonia-oxidizing archaea	Nitrogen cycling, stress-associated nutrient turnover	Nutrient availability under stress	Offre et al. (2013), Prosser (2012)
Protists	Bacterivorous protists	Selective bacterivory, regulation of microbial turnover	Nutrient mineralization, community succession	Liao et al. (2024)
Viruses (bacteriophages)	Phages	Lytic and lysogenic interactions, horizontal gene transfer	Microbial population control, functional stability	Chevallereau et al. (2022)
Microbial BCAs	*Trichoderma, Bacillus, Pseudomonas*	Targeted pathogen suppression, microbiome modulation	Disease control, resilience enhancement	Compant et al. (2019), De Faria et al. (2021)

### 2.3 Functional roles in plant health and soil processes

The rhizosphere microbiome contributes to plant health through a set of interrelated functional processes that collectively influence nutrient availability, immune competence, and stress tolerance. Facilitation of nutrient acquisition is a core function, particularly under low-input or stress-prone conditions (De Faria et al., 2021). Diverse microbial taxa including *Bacillus*, *Pseudomonas*, *Trichoderma*, and AMF enhance the bioavailability of key nutrients such as phosphorus, potassium, and zinc through mineral solubilization, enzymatic activity, and chelation mechanisms (Ansabayeva et al., 2025; Thepbandit and Athinuwat, 2024). In parallel, diazotrophic bacteria such as *Azospirillum*, *Burkholderia*, and *Rhizobium* contribute to nitrogen input via biological fixation, supporting plant nutrition in resource-limited systems (Imran et al., 2021).

Beyond nutrient-related functions, rhizosphere microorganisms modulate plant physiological responses through hormonal and immune-mediated pathways. Many bacteria and fungi influence plant development by producing or regulating phytohormones, including indole-3-acetic acid (IAA), cytokinins, gibberellins, and ACC deaminase, thereby affecting root architecture and growth dynamics (Kaya, 2024; Orozco-Mosqueda et al., 2023). These microbial effects are frequently linked to improved water-use efficiency, enhanced nutrient uptake, and increased tolerance to abiotic stress. In addition, specific members of the rhizosphere microbiome can activate induced systemic resistance (ISR) by modulating jasmonic acid (JA) and ethylene (ET) signalling pathways, activating broad-spectrum defense without the growth penalties often associated with systemic acquired resistance (SAR) (Rabari et al., 2023; Zhu et al., 2022). Well-characterized ISR-inducing taxa include *Pseudomonas fluorescens*, *Bacillus subtilis*, and *Trichoderma harzianum* (Panpatte et al., 2016; Lee Díaz et al., 2021).

The rhizosphere microbiome also contributes to disease suppression through both direct antagonism and community-level interactions. Antagonistic microbes inhibit pathogens via competition for resources, mycoparasitism, and the production of antimicrobial metabolites such as lipopeptides, volatile organic compounds, and hydrolytic enzymes (Compant et al., 2019; Iqbal et al., 2023). Importantly, pathogen suppression often emerges from the collective activity of stable microbial consortia rather than from individual taxa. Disease-suppressive soils exemplify this principle, as they are consistently associated with enrichment of microbial groups such as *Pseudomonas*, *Trichoderma*, and *Streptomyces*, which constrain pathogens including *Fusarium oxysporum* and *Rhizoctonia solani* through antibiotic production and competition for iron (Table 1; De Faria et al., 2021; Mazzola, 2002). In addition to biotic interactions, rhizosphere microorganisms play a key role in mitigating abiotic stress. By enhancing antioxidant capacity, osmotic adjustment, and stress-responsive gene expression, beneficial microbes can improve plant tolerance of salinity, drought, and temperature extremes (Khan et al., 2021; Sharma et al., 2020). However, the magnitude and consistency of these benefits are strongly context-dependent, reflecting interactions between microbial composition, host genotype, and environmental conditions.

### 2.4 Sensitivity to abiotic and biotic stresses

Despite its ecological adaptability, the rhizosphere microbiome is highly sensitive to environmental disturbance, with abiotic and biotic stressors often interacting to destabilize community structure and function. Abiotic stresses such as drought, salinity, pH fluctuations, and thermal extremes can reduce microbial diversity, suppress keystone taxa, and compromise microbiome-mediated processes that support plant nutrition and defense (Khan et al., 2023; Munir et al., 2022). For example, drought frequently reduces the abundance of beneficial plant-associated taxa such as *Pseudomonas* and *Bacillus* while favoring desiccation-tolerant groups like Actinobacteria (Santos-Medellín et al., 2017). Similarly, reduced soil moisture availability can limit colonization by AMF, despite its importance for plant water-use efficiency (Abdalla et al., 2023). Salinity stress further constrains microbial richness and disrupts symbiotic nutrient acquisition, particularly in non-halophilic taxa, through osmotic and ionic toxicity (Andronov et al., 2012; Munir et al., 2022).

Biotic stressors, particularly soilborne pathogens, also exert strong selective pressures on rhizosphere communities. Pathogen invasion by species such as *F. oxysporum*, *F. solani*, *Ralstonia solani*, and *R. solani* can restructure microbial networks, suppress beneficial microbes, and induce community dysbiosis (Table 1; Deng et al., 2021; Kashyap et al., 2023; Su et al., 2020). While pathogens may outcompete resident taxa through allelopathy, niche displacement, or immune manipulation, plants can partially counteract these effects through a “cry-for-help” response, releasing targeted exudates that recruit protective microbes (Berendsen et al., 2012; Rolfe et al., 2019). This response highlights the dynamic and context-dependent nature of plant–microbiome interactions under disease pressure.

Climate change exacerbates the complexity of these interactions by increasing the frequency and intensity of compound stress events such as simultaneous drought and pathogen attack. These multifactorial pressures can undermine both plant immunity and microbiome resilience, thereby reducing the rhizosphere’s buffering capacity against further disturbance (Choudhary and Senthil-Kumar, 2024). In this context, microbiome resilience reflects the capacity of the rhizosphere community to maintain or rapidly recover key structural and functional attributes following stress. Elevated CO₂ and temperature can further alter root exudation patterns by modifying plant carbon allocation and metabolic activity, reshaping microbial recruitment, and favoring stress-tolerant or opportunistic taxa over beneficial symbionts (De Faria et al., 2021; Li et al., 2021). Anthropogenic inputs including pesticides and excessive fertilizer treatment may exacerbate these effects by degrading mutualistic networks and simplifying community composition (Jacobsen and Hjelmsø, 2014). Consequently, sustaining a functionally resilient rhizosphere microbiome depends not only on microbial diversity but also on network redundancy, host compatibility, and the legacy effects of agronomic practices. The development of climate-smart adaptive agroecosystems thus requires knowledge of the thresholds at which beneficial functions collapse and the availability of strategies to restore them through inoculants, amendments, or host breeding.

## 3 Microbial BCAs: key players and mechanisms

Microbial BCAs are a promising class of eco-compatible tools for disease suppression and sustainable crop production. Among the most widely studied genera are *Trichoderma*, *Clonostachys*, *Bacillus*, and *Pseudomonas*, all of which exhibit broad antagonistic activity against phytopathogens and contribute to enhanced plant health under both biotic and abiotic stress (Guzmán-Guzmán et al., 2019; Karlsson et al., 2015; Lahlali et al., 2022). While their beneficial effects have mainly been demonstrated under controlled or semi-controlled conditions, these genera exhibit high environmental adaptability and root colonization efficiency, and have multifunctional roles that extend beyond pathogen antagonism.

The core mechanisms by which BCAs act against microbial phytopathogens include antibiosis, ISR, mycoparasitism, and resource competition (Table 2). Antibiosis involves the secretion of antimicrobial metabolites such as lipopeptides, antibiotics, siderophores, and hydrolytic enzymes that inhibit or lyse pathogenic cells. For instance, *B. subtilis* produces surfactins and iturins that disrupt fungal membranes, while *P. fluorescens* is known for its production of phenazine and pyoluteorin compounds with broad-spectrum antifungal activity (Fischer et al., 2013). *Trichoderma* species are particularly effective in this regard, releasing cell wall-degrading enzymes such as chitinases and glucanases alongside volatile organic compounds that suppress a wide array of soilborne fungi (Debode et al., 2018; Guzmán-Guzmán et al., 2019). However, the production, stability, and bioavailability of these metabolites are highly context-dependent. These processes are influenced by factors such as soil nutrient status, pH, and competitive pressure from indigenous microbial communities, which often constrains their reproducibility under field conditions.

**Table 2:** Representative microbial biocontrol agents (BCAs), their dominant mechanisms of action, and reported effects on rhizosphere microbiome structure and plant performance.

BCA group/genus (examples)	Primary mechanisms of action	Reported microbiome-level effects	Plant/stress-related outcomes	References
*Clonostachys* and *Trichoderma* spp.	Mycoparasitism, ISR, enzyme secretion	Shifts toward beneficial fungal taxa, increased network connectivity	Disease suppression, drought tolerance	Karlsson et al. (2015), Debode et al. (2018)
*Bacillus* spp.	Antibiosis, ISR, competition	Shifts in bacterial community composition, enrichment of PGPR-associated taxa	Pathogen control, improved growth	Compant et al. (2019), Fischer et al. (2013), Rabari et al. (2023)
*Pseudomonas* spp.	Antibiotic production, siderophores, ISR	Suppression of pathogens, altered rhizosphere structure	Disease resistance, nutrient uptake	Lahlali et al. (2022), Mazzola (2002)
Microbial consortia	Functional complementarity among microbial members	Increased microbial diversity and stability	Enhanced stress resilience	Vishwakarma et al. (2020), Mamun et al. (2024)

Mycoparasitism, a direct parasitic interaction in which a biocontrol fungus invades and degrades a pathogenic one, is well-documented in *T. harzianum* and *Clonostachys rosea* (Chet, 1987; Karlsson et al., 2015). These fungi recognize pathogen-specific molecular patterns, initiate targeted enzymatic degradation, and physically coil around or penetrate pathogen hyphae, leading to pathogen death. This process is often coupled with competition for space and nutrients in the rhizosphere, effectively excluding pathogens from becoming established on the root surface. Despite its favorable effects in controlled systems, effective mycoparasitism in dynamic soil environments requires the establishment and persistence of the mycoparasitic agent in the soil, which is often difficult to achieve with introduced BCAs. In addition to their antagonistic functions, some BCAs play an important role in priming plant defense responses. Many strains induce ISR, a plant-mediated immune enhancement triggered by microbial signals such as lipopolysaccharides or flagellins (Pieterse et al., 2014). Unlike SAR, ISR typically does not involve accumulation of salicylic acid (SA) or pathogenesis-related (PR) proteins, allowing plants to maintain growth while remaining primed for faster and stronger defense upon pathogen attack (Heil and Bostock, 2002). Emerging evidence also highlights the role of microbial BCAs in improving plant tolerance to abiotic stress, a less explored but increasingly relevant function under climate change scenarios. For example, *Trichoderma* spp. can improve water-use efficiency, enhance antioxidant activity, and modulate stress-responsive genes, thereby mitigating the adverse effects of drought and salinity (Sorahinobar et al., 2025). Similarly, *Bacillus* spp. have been shown to promote root growth and osmolyte accumulation under heat and osmotic stress conditions (Nivetha et al., 2024). However, much of this evidence derives from laboratory or greenhouse experiments, and the desired beneficial responses are not consistently observed in heterogeneous field environments. Further study is thus needed to clarify the molecular and physiological pathways underpinning these benefits in complex, multi-stress field environments, and to evaluate their quantitative impacts on yields under severe stress.

## 4 Interactions between microbial BCAs and the rhizosphere microbiome

### 4.1 Microbiome modulation by microbial BCAs

The introduction of microbial BCAs into the rhizosphere to suppress phytopathogens also modulates native microbial communities via both direct and indirect mechanisms that influence community interactions and stability. These mechanisms include niche competition, secretion of antibiotics, interference with microbial signalling pathways, and metabolic cross-feeding, wherein one microbe utilizes metabolites produced by another (Table 2; Tang et al., 2025; Zhang et al., 2023). In addition to these direct microbial interactions, BCAs can influence rhizosphere community assembly through plant-mediated effects. BCA inoculation has been shown to modify the volume and composition of root exudates, for example by changing the content of sugars, organic acids, amino acids, and secondary metabolites, which in turn shape microbial recruitment and activity in the rhizosphere. Such exudate-mediated shifts can selectively promote beneficial taxa or alter competitive interactions among resident microbes, thereby indirectly contributing to microbiome restructuring and functional outcomes (Berendsen et al., 2012; Haichar et al., 2008; Rolfe et al., 2019). Through these processes, BCAs influence microbial diversity, including both alpha diversity (within-sample richness) and beta diversity (between-sample compositional differences). The magnitude and direction of these shifts depend on the host plant and soil type as well as the resident microbiota (Debode et al., 2018; Wilson and Arunachalam, 2024). Inoculation with BCAs can thus act as an ecological perturbation, triggering shifts in community structure and ecosystem function. However, such modulation is not universally beneficial. In cases of niche overlap or ecological antagonism, BCAs may competitively displace native microbial taxa crucial for nutrient cycling or stress buffering. This can potentially trigger cascading nontarget effects across the rhizosphere microbiome (Pearson and Callaway, 2003, 2005; Trabelsi and Mhamdi, 2013). Additionally, BCAs fail to persist in some settings due to poor compatibility with the indigenous microbiome or unfavorable soil conditions, reducing their long-term efficacy (Schulz et al., 2019).

### 4.2 Factors influencing inoculant performance

The effectiveness of BCAs in shaping rhizosphere microbiomes is highly variable, particularly when comparing laboratory, greenhouse, and field settings, reflecting the interplay of abiotic, biotic, and ecological factors. Soil physicochemical traits such as pH, texture, nutrient availability, and organic matter content are key abiotic drivers that strongly influence microbial colonization and functional expression (Berg and Smalla, 2009; Trabelsi and Mhamdi, 2013). For instance, acidic soils can limit *Bacillus* spp. sporulation, while organic-rich soils may reduce the competitive advantage of nutrient-solubilizing strains. Beyond effects on colonization, abiotic conditions can directly regulate the expression of BCA functions including the biosynthesis and stability of antibiotics and other secondary metabolites, thereby modulating antagonistic activity independently of population size (Berg and Smalla, 2009; Raaijmakers and Mazzola, 2012). Such context-specific responses underscore the challenge of developing universally effective formulations.

Plant genotype exerts an equally strong filtering effect. Root exudate chemistry, architectural traits, and immune signalling not only shape microbial recruitment but also determine compatibility with inoculated strains. Genotype-specific filtering can facilitate or block BCA establishment depending on how introduced microbes overlap with resident microbiota niches (Philippot et al., 2013; Rawal et al., 2024). This highlights the need to consider crop genetic background in inoculant design, particularly in breeding programs that historically overlook plant–microbe interactions (Iqbal et al., 2025). Climatic variability adds another layer of complexity. Temperature extremes, soil moisture fluctuations, and seasonal cycles modulate microbial persistence and activity. For example, *B. subtilis* and *T. harzianum* have shown strong antagonism in controlled trials but exhibit inconsistent or diminished efficacy under field conditions, suggesting that environmental complexity often limits the translation of laboratory efficacy (Rigobelo et al., 2024).

Colonization and persistence are critical bottlenecks. Many inoculants fail to establish durable populations due to competitive exclusion by native microbiota, predation by protists, or suboptimal timing and delivery methods. In addition to effects on establishment, interactions with indigenous microbial communities can directly constrain or enhance BCA efficacy through interspecies metabolic interference or facilitation. For example, *Stenotrophomonas maltophilia* can degrade lipopeptide antibiotics produced by *Bacillus* spp. (e.g., iturin, fengycin, surfactin), thereby attenuating biocontrol of tomato bacterial wilt despite successful BCA colonization (Peng et al., 2025). In contrast, positive interactions with resident microbial keystone taxa can enhance BCA performance. A recent study showed that specific indigenous microbes stimulated antibiotic biosynthesis in *Streptomyces*, leading to improved disease suppression through metabolite-mediated induction rather than direct antagonism alone (Sun et al., 2025). These contrasting outcomes highlight that BCA efficacy is highly context-dependent rather than universally reproducible. Even when initial colonization is achieved, BCA-mediated functions such as ISR or mycoparasitism are often regulated by density-dependent microbial signalling and plant-derived cues, which may not be consistently activated across diverse soil environments (Moussa and Iasur Kruh, 2025). Moreover, traits observed during *in vitro* screening often fail to translate to complex rhizosphere environments, where multi-trophic interactions, fluctuating nutrient availability, and stress events shape microbial behavior. This discrepancy illustrates the limitations of current screening pipelines, which often neglect ecological compatibility and stress resilience.

### 4.3 Risks and trade-offs

Despite their potential, BCAs are not without risks. One critical concern is the displacement of native beneficial microbes, particularly in systems with already well-functioning microbial communities. The introduction of dominant inoculants can reduce microbial diversity or suppress indigenous taxa that play critical roles in nutrient cycling, pathogen suppression, and stress buffering (Pearson and Callaway, 2003; Trabelsi and Mhamdi, 2013). Such non-target effects may compromise essential ecosystem functions if introduced BCAs competitively displace resident microbes that are functionally important but not directly associated with disease suppression. These ecological trade-offs are especially evident in monospecific inoculations, which often lack the resilience and functional redundancy characteristic of native microbial consortia. Many BCAs also exhibit narrow-spectrum efficacy, performing effectively against specific pathogens under controlled conditions but showing limited broad-spectrum protection and consistency in complex field environments (Leal et al., 2024).

Beyond these general constraints, more subtle ecological risks may arise. Targeting a single pathogen can inadvertently lead to ecological release of secondary or opportunistic pathogens, thereby unpredictably altering disease complexes. Unintended effects may also result from horizontal gene transfer, interference with microbial signalling networks, or suppression of keystone taxa that support the rhizosphere’s structural stability. Repeated or high-frequency BCA applications may amplify these effects by cumulatively reshaping microbial networks and selecting for altered interaction dynamics over time. Such perturbations can compromise microbiome resilience, reduce invasion resistance, and impair ecosystem services that underpin long-term soil health. Importantly, these risks are context-dependent: BCAs may enhance resilience by restoring lost functions in degraded or simplified systems but could disrupt established microbial equilibria in biodiverse or organically managed soils. Despite these concerns, there have been few long-term evaluations of the ecological consequences of repeated BCA applications across cropping cycles and environmental conditions. Current risk assessment approaches are largely oriented toward short-term efficacy, with less emphasis on microbiome-level impacts, biosafety considerations, or cumulative ecological effects, highlighting the need for more holistic and longitudinal perspectives on the sustainability of microbial biocontrol interventions.

## 5 Climate-responsive microbial biocontrol

### 5.1 Fungal pathogens and climate change

Climate change is reshaping the global epidemiology of fungal diseases, with increasing incidences attributed to drought, salinity, temperature extremes, and shifting precipitation patterns (Bebber et al., 2013; Singh et al., 2023). These stressors extend the geographic range of pathogens such as *Fusarium* spp., *Verticillium* spp., and *Botrytis cinerea*, while simultaneously enhancing their aggressiveness and survival under adverse conditions.

Plant susceptibility is aggravated under abiotic stress. Drought impairs root development, alters rhizodeposition patterns, and limits the plant’s ability to recruit beneficial microbes. These factors collectively increase plants’ vulnerability to soilborne pathogens (Preece and Peñuelas, 2016; Zahra et al., 2023). Likewise, salinity and high temperatures have been shown to downregulate key immune signalling pathways, including those regulated by SA, JA, and ET, which are essential for SAR and induced defense responses (Rossi et al., 2023). Importantly, interactions between abiotic and biotic stressors are often synergistic rather than additive. Combined stress conditions, such as drought coinciding with a pathogen challenge, can lead to greater physiological damage than either stress alone. This is partly due to compounded suppression of plant immune responses and microbiome-mediated defense mechanisms. These interactions also reduce the functional stability of rhizosphere microbial communities, which limits their capacity to buffer against pathogen invasion (Ramegowda and Senthil-Kumar, 2015; Suzuki et al., 2014).

### 5.2 Microbial BCA strategies for climate resilience

Microbial BCAs provide two key functions under climate stress conditions: they suppress phytopathogens while simultaneously enhancing plant tolerance of abiotic stressors (Sheoran et al., 2025). These functions are mediated by stressor-specific mechanisms that operate at both the cellular and rhizosphere ecosystem levels and are linked to key climate variables including drought-induced water limitation, temperature-driven metabolic constraints, and salinity-associated osmotic stress. However, these benefits are not consistently observed across experimental scales: desirable outcomes are seen more frequently under controlled conditions than in heterogeneous field environments. Understanding and optimizing these interactions will be essential for developing climate-resilient cropping systems. Representative studies demonstrating BCA-induced microbiome and plant responses under stress conditions are summarized in Table 3.

**Table 3:** Representative studies demonstrating microbial biocontrol agents (BCAs)–induced changes in microbiome structure and plant responses under stress conditions relevant to climate resilience.

Stress condition	BCA system (example)	Observed microbiome/plant response	Level of response	References
Drought	*Trichoderma* spp.	Enrichment of stress-tolerant taxa; enhanced antioxidant activity	Microbiome + plant	Mona et al. (2017)
Salinity	PGPR (*Bacillus*, *Pseudomonas*)	Osmolyte accumulation; improved ion homeostasis	Plant physiology	Ha-Tran et al. (2021)
Drought/salinity	PGPR	Microbial community restructuring; improved yield stability	Microbiome + plant	Al-Turki et al. (2023)
Multi-stress	Microbial consortia	Increased community stability and functional redundancy	Microbiome	Mamun et al. (2024)

#### 5.2.1 Stress-responsive modulation by BCAs

Fungal BCAs, particularly *Trichoderma* spp., have a well-documented capacity to mitigate drought- and heat-associated stress through coordinated modulation of plant antioxidant systems, osmotic balance, and hormonal pathways (Geng et al., 2025). In maize and tomato, inoculation with *T. asperellum* and *T. harzianum* significantly enhanced the expression and activity of key antioxidant enzymes such as superoxide dismutase (SOD), catalase (CAT), and ascorbate peroxidase (APX), which all play important roles in detoxifying reactive oxygen species (ROS) generated under water-deficit conditions (Mona et al., 2017; Yousaf et al., 2023). Such biochemical priming reduces oxidative cellular damage, stabilizes membrane integrity, and improves photosynthetic efficiency primarily in laboratory and greenhouse studies. Moreover, plant growth-promoting rhizobacteria (PGPR) such as *P. fluorescens*, *Azospirillum brasilense*, and *B. subtilis* have demonstrated the capacity to improve plant performance under drought- and salinity-driven osmotic stress (Table 3). These bacteria modulate the synthesis and accumulation of osmolytes such as proline, trehalose, and glycine betaine, which facilitate cellular osmotic adjustment and help maintain turgor pressure under dehydrating conditions (Ha-Tran et al., 2021). Several PGPR strains produce exopolysaccharides (EPS) that enhance soil aggregation and improve water-holding capacity, partially offsetting moisture limitation in drought-prone soils (Zheng et al., 2018). Moreover, some strains trigger phytohormonal crosstalk by producing analogues or regulators of IAA, abscisic acid (ABA), and ET, thereby influencing root system architecture and stomatal conductance (Khan et al., 2020). This hormonal tuning facilitates nutrient uptake under stress and also contributes to adaptive regulation of plant water use under variable climatic conditions. These microbial mechanisms represent promising bio-based tools for buffering crops against increasingly frequent climate-induced stressors.

#### 5.2.2 Mechanistic insights from omics technologies

Advances in omics technologies have provided valuable insights into the mechanistic basis of microbe-mediated stress tolerance in plants under specific climate-related stressors. Transcriptomic analyses of plants inoculated with *Trichoderma* spp. revealed the upregulation of stress-responsive genes involved in aquaporin regulation, expression of late embryogenesis abundant (LEA) proteins, and ABA signalling, among other things. These changes collectively contribute to improved water retention, membrane protection, and stomatal regulation under drought conditions (Doni et al., 2019; Zhang et al., 2022). At the molecular level, these adjustments show how fungal BCAs can fine-tune host stress physiology in addition to performing their traditional biocontrol roles. Metabolomic studies have similarly shown that PGPR elicit the synthesis of osmo-protectants such as proline and glycine betaine as well as secondary metabolites such as flavonoids and phenolics that function as ROS scavengers and cell protectants during abiotic stress (Al-Turki et al., 2023). In salt-stressed tomato and soybean plants, PGPR-induced metabolic reprogramming has been linked to improved ion homeostasis, photosynthetic stability, and yield retention (Al-Turki et al., 2023; Kumawat et al., 2023). Amplicon-based microbiome profiling (16S rRNA and ITS sequencing) has demonstrated that BCAs can also restructure rhizosphere microbial networks, promoting the enrichment of drought-resilient taxa such as Actinobacteria and certain classes of Proteobacteria while reducing the relative abundance of opportunistic pathogens (Gao et al., 2023). These shifts reflect functional selection for stress-tolerant guilds, potentially enhancing microbial synergy and functional redundancy under environmental perturbations. However, it should be noted that most omics-based insights derive from controlled systems, and their predictive value for field-scale resilience remains limited.

#### 5.2.3 Role of microbial consortia and formulations

Microbial consortia are mixtures of multiple co-inoculated bacterial and/or fungal strains. It is increasingly recognized that such consortia can offer greater stability and multifunctionality than single strain inoculations under climate-induced stress conditions. Unlike monospecific BCAs, consortia can exploit functional complementarity wherein different microbes perform distinct but synergistic roles such as nutrient solubilization, pathogen suppression, and hormone modulation (Vishwakarma et al., 2020). The combined application of *T. harzianum*, *B. subtilis*, and AMF in tomato, strawberry, and potato systems enhanced drought tolerance and disease suppression, which was attributed to co-regulation of ABA signalling, antioxidant enzyme activation, and root colonization efficiency (Mamun et al., 2024; Minchev et al., 2021). However, consistent performance of consortia is not guaranteed: antagonism among consortium members or competition with resident microbiota can negate expected benefits if ecological compatibility is not adequately assessed. Formulation technologies are another important determinant of field efficacy. Encapsulation in biopolymeric matrices such as alginate beads, biochar, or nanocarriers has been shown to enhance microbial viability under environmental stress by buffering against UV radiation, desiccation, and oxidative stress (Balla et al., 2022; Cruz-Barrera et al., 2024). Even so, formulation success remains highly context-dependent and delivery systems must be tailored to specific soil textures, pH ranges, and moisture regimes in order to optimize BCA release kinetics and colonization dynamics in climate-challenged regions.

## 6 Case studies in crop systems: microbiome and biocontrol interactions

The following case studies illustrate how interactions between microbial BCAs and the rhizosphere microbiome manifest across diverse crop systems and stress contexts. While each example highlights crop-specific outcomes, they collectively reveal recurrent patterns in microbiome modulation, alongside pronounced context dependence driven by host traits, environmental conditions, and management practices. Table 4 presents a synthesis comparing the features of these systems and highlighting key divergences.

**Table 4:** Comparative synthesis of microbial biocontrol agents (BCAs)–microbiome interactions across crop systems.

Crop system	Representative BCAs	Dominant stress context	Microbiome-level effects	Plant-level outcomes	Key sources of variability	References
Strawberry	*Trichoderma*, *Bacillus*, *Aureobasidium*	Soilborne pathogens; postharvest stress	Increased diversity, enhanced network connectivity, enrichment of keystone taxa	Disease suppression, improved fruit quality	Baseline microbiome composition, delivery method	Debode et al. (2018), Iqbal et al. (2021), Kang et al. (2025), Menéndez-Cañamares et al. (2024)
Tomato/Cucurbits	*Bacillus*, *Pseudomonas*, *Trichoderma*	Pathogen pressure; drought; salinity	Shifts in bacterial–fungal balance, AMF enrichment	Disease control, stress tolerance	Host genotype, soil type, stress intensity	Ali et al. (2025), Mehmood et al. (2023), Moussa and Iasur Kruh (2025)
Grapevine/Banana	*Trichoderma*, *Bacillus*	Chronic pathogen pressure; drought	Increased functional redundancy, endosphere restructuring	Reduced disease severity, yield stability	Crop perenniality, climate variability	Chen et al. (2024), Damodaran et al. (2020), Di Marco et al. (2004)
Rice/Maize	Microbial consortia (N-fixers, P-solubilizers, BCAs)	Salinity; temperature stress	Functional complementarity, enhanced nutrient cycling	Biomass and yield maintenance	Cultivar compatibility, land-use history	Marzouk et al. (2025), Sackey et al. (2025), Tyagi et al. (2023)

### 6.1 Strawberry as a model system

Strawberry (*Fragaria* × *ananassa*) has emerged as a valuable model for studying interactions between microbial BCAs and rhizosphere microbiome dynamics due to its relatively short lifecycle, well-characterized microbial baseline, and high sensitivity to biotic and abiotic stresses (Debode et al., 2018; Kang et al., 2025). Its vulnerability to soilborne pathogens including *B. cinerea*, *F. oxysporum*, and *Colletotrichum acutatum*, combined with a microbiome that is both diverse and responsive to disturbance, provides a high-resolution framework for testing microbiome-modulating interventions (Kang et al., 2025). Inoculation with BCAs such as *T. harzianum*, *Aureobasidium pullulans*, and *B. subtilis* have consistently suppressed *B. cinerea* and *C. acutatum* while simultaneously enriching rhizosphere diversity and microbial network connectivity in strawberry studies (Freeman et al., 2004; Huang et al., 2023; Iqbal et al., 2021, 2023; Mannaa et al., 2023; Menéndez-Cañamares et al., 2024). These BCAs suppress pathogens via both indirect antagonistic mechanisms, including antibiosis, and direct interactions such as mycoparasitism. They also appear to facilitate beneficial shifts in core microbial taxa associated with nutrient mobilization, systemic resistance, and root colonization. Bee-vectored delivery of *C. rosea* and *A. pullulans* has demonstrated dual benefits, enhancing pre-harvest disease resistance and improving post-harvest fruit quality by extending shelf life and reducing decay incidence (Iqbal et al., 2022; Kumar et al., 2024). These strategies showcase systems-level integration of biocontrol, highlighting its potential application across plant developmental stages and the agro-supply chain. Importantly, in strawberry systems, BCA inoculation has been shown to alter the rhizosphere fungal community beyond direct pathogen suppression, including changes in community composition and associated plant defense responses (Debode et al., 2018).

### 6.2 Tomato, muskmelon, and cucumber: managing pathogen pressure and abiotic stress

Tomato (*Solanum lycopersicum*), muskmelon (*Cucumis melo*), and cucumber (*Cucumis sativus*) have been important systems for evaluating the dual role of microbial BCAs in disease suppression and abiotic stress mitigation. In tomato, complex microbial consortia comprising *P. fluorescens*, *T. asperellum*, *B. velezensis*, and *B. subtilis* have shown efficacy against a spectrum of soilborne pathogens, including *R. solanacearum*, *F. oxysporum* f.sp. *lycopersici*, and *Meloidogyne* spp. (root-knot nematodes) (Ali et al., 2025; Gu et al., 2023; Moussa and Iasur Kruh, 2025; Obiazikwor et al., 2025; Wan et al., 2017). These BCAs not only provided biocontrol through direct antagonism and ISR induction but also reshaped the microbial composition of the rhizosphere, shifting patterns of alpha and beta diversity. Importantly, BCA application was associated with a higher abundance of Actinobacteria and mycorrhizal fungi such as *Glomus* spp., which are known to contribute to enhanced phosphorus solubilization, root colonization, and pathogen exclusion (Gu et al., 2023; Wan et al., 2017).

Promising results have also been obtained in cucurbit systems, particularly muskmelon and cucumber. Applications of *B. amyloliquefaciens*, *B. subtilis*, and *Pseudomonas chlororaphis* reduced the incidence of *F. oxysporum* f. sp. *radicis-cucumerinum*, *Podosphaera xanthii*, *B. cinerea*, *R. solani* and *Pythium* spp., especially under drought and saline conditions in which chemical controls often fail (Mehmood et al., 2023; Ni and Punja, 2020). The BCAs also activated host stress responses including the accumulation of antioxidant enzymes (e.g., SOD, CAT, APX) and compatible solutes (e.g., proline, glycine betaine), thus contributing to osmotic adjustment and oxidative damage mitigation. These results emphasize that effective biocontrol is not limited to pathogen suppression but often coincides with improved rhizosphere functionality and resilience.

### 6.3 Grapevine and banana: trunk disease and wilt suppression

Perennial crops such as grapevine (*Vitis vinifera*) and banana (*Musa* spp.) are high-value systems that are increasingly threatened by persistent fungal pathogens and environmental stressors. In grapevines, trunk diseases caused by *Phaeomoniella chlamydospora* and *Eutypa lata* have shown significant responsiveness to treatment with *T. atroviride*, *T. harzianum* and *B. subtilis*, especially in Mediterranean climates characterized by thermal variability and drought stress (Di Marco et al., 2004; Gramaje et al., 2018; Kotze et al., 2011; Mesguida et al., 2023). These BCAs reduced disease severity while also inducing restructuring of both rhizosphere and endosphere microbial communities. This led to increased functional redundancy in bacterial guilds associated with xenobiotic degradation, biosynthesis of phytohormones including auxins and gibberellins, and immune modulation, suggesting enhanced ecological stability under pathogen pressure (Chen et al., 2024).

In bananas, inoculation with *T. asperellum*, *T. reesei*, and *T. koningiopsis* achieved partial control of *F. oxysporum* f. sp. *cubense* tropical race 4 (Foc TR4), a pathogen of global concern (Damodaran et al., 2020; Ploetz, 2015). Field studies across Asia and Latin America showed consistent suppression of disease symptoms alongside improved yield metrics. Treated plants exhibited elevated expression of PR-related genes, improved root architecture, and an increase in rhizospheric populations of plant growth-promoting bacteria (PGPB), contributing to both biotic resistance and physiological resilience (Mon et al., 2021).

### 6.4 Rice, maize, and other staples: consortia and microbiome modulation

Cereal staples such as rice (*Oryza sativa*) and maize (*Zea mays*) are increasingly being targeted for treatment with microbial consortia to enhance stress resilience and nutrient efficiency under climate stress (Jha et al., 2013; Tyagi et al., 2023; Zayadan et al., 2014). These consortia typically combine functionally complementary taxa like nitrogen-fixers (*Azospirillum*, *Serratia*), phosphate-solubilizers (*Azotobacter, Bacillus*, *Enterobacter*), and biocontrol fungi (*Trichoderma* spp.) to simultaneously address multiple agroecological constraints (Altomare et al., 1999; De Vries and Wallenstein, 2017; Hermosa et al., 2013; Kong et al., 2018; Rizvi and Khan, 2018; Woo and Pepe, 2018). Under both controlled and field environment, such inoculants have been shown to improve crop performance, particularly in low-input systems exposed to salinity or thermal extremes. Mechanistically, these benefits are linked to enhanced ion homeostasis (e.g., improved K^+^/Na^+^ ratios), modulation of antioxidant enzyme activity, and reinforcement of root–soil interactions through exopolysaccharide production and root surface colonization. In salt-affected paddy systems, inoculated rice plants exhibited reduced sodium uptake, enhanced osmolyte accumulation, and improved nutrient use efficiency, allowing biomass yields to be maintained without synthetic inputs (Marzouk et al., 2025; Sackey et al., 2025; Saleem et al., 2025). However, the performance of these consortia remains highly variable. Inoculant efficacy depends on cultivar compatibility, soil physicochemical properties, and previous land-use history, which collectively shape the recruitment, colonization, and functional integration of applied microbes. However, most studies have focused on short-term growth and yield outcomes, paying limited attention to long-term microbiome restructuring, ecological trade-offs, or economic scalability.

## 7 Challenges and limitations of microbial BCAs

Despite the expanding role of microbial BCAs in sustainable agriculture, their inconsistent performance under field conditions remains a major problem. Laboratory and greenhouse trials often yield promising results, but efficacy frequently declines in open-field systems due to the combined effects of fluctuating soil physicochemical properties, climatic variability, and the ecological complexity of indigenous microbiota. Context-dependent interactions between the inoculant, host genotype, and native microbial networks can either facilitate or hinder colonization, while abiotic stresses such as drought and salinity further compromise microbial viability and plant–microbe signalling.

An important challenge is the short-lived colonization of many BCAs, including widely used agents such as *Trichoderma* and *Bacillus* spp. Competitive exclusion by resident microbiota, predation by protists, and suboptimal environmental cues often prevent long-term persistence. Under such conditions, many protective traits, including mycoparasitism, antibiosis, and ISR are regulated by density-dependent microbial signalling, secondary metabolite production, and host-derived signalling cascades. Consequently, incomplete establishment can render BCAs functionally ineffective. While multi-strain consortia and synthetic microbial communities (SynComs) offer a promising avenue to improve stability and broaden functional capacity, mechanistic understanding of interspecies interactions remains limited. Synergistic combinations can enhance disease suppression and stress tolerance, yet antagonistic effects or suppression by native soil communities are also possible. Moreover, the interplay between consortium performance and abiotic stress adaptation is poorly characterized, constraining the development of climate-resilient inoculants.

Regulatory and adoption barriers further limit BCA deployment. Registration frameworks are often fragmented and slow to adapt to living, strain-specific products, especially for consortia or engineered microbes. In many regions, inconsistent biosafety evaluation, quality control, and shelf-life standards undermine both market entry and farmer confidence. Adoption is further constrained by limited farmer awareness, inadequate technical support, and the perception that BCAs are less reliable than synthetic pesticides under high disease pressure or adverse weather.

## 8 Conclusions and future directions

As agriculture transitions toward sustainable and climate-resilient production systems, microbial BCAs and rhizosphere microbiome-based interventions are expected to become integral components of crop protection and productivity frameworks. Realizing this potential will require a shift from unsystematic inoculation practices toward precision-designed, ecologically compatible, and context-specific microbial solutions. Rationally assembled SynComs offer significant advantages over conventional single-strain inoculants through the integration of complementary functions, including nitrogen fixation, phosphate solubilization, siderophore production, and stress-induced priming. This functional integration also confers redundancy and ecological stability under variable field conditions. Advances in high-throughput omics, including metagenomics, transcriptomics, and metabolomics, now enable the identification of microbial candidates based on functional traits, stress-resilience genes, and biosynthetic potential, rather than cultivability alone. Coupled with genome editing and synthetic biology, these approaches could accelerate the development of functionally optimized BCAs, tailored to specific soil types, climatic regimes, and crop genotypes. However, approaches relying on synthetic biology, genome editing, and fully engineered SynComs largely represent longer-term opportunities, as their field validation, regulatory approval, and ecological risk assessment remain ongoing challenges.

Integration of BCAs into microbiome-informed IPM frameworks will be essential to ensure both disease suppression and long-term ecosystem functionality. Microbiome-informed biological control emphasizes the selection and deployment of BCAs based on key functional traits including stress tolerance, niche complementarity, persistence under field conditions, and the capacity to interact synergistically with host plants and resident microbiomes. In the near term, strategies such as trait-based strain selection, formulation optimization, and the integration of BCAs into existing IPM programs offer more immediately feasible pathways for improving disease management and stress resilience. Emerging precision agriculture tools such as real-time microbiome diagnostics, drone-assisted delivery, and GPS-guided application can enable site-specific, optimized deployment that maximizes efficacy while minimizing non-target impacts. Equally, leveraging the plant holobiome concept in breeding programs offers a path toward host genotypes inherently predisposed to recruit and sustain beneficial microbiota under stress. Embedding microbiome responsiveness into genetic selection criteria, could improve future crops’ colonization efficiency and functional engagement with BCAs, fostering durable resilience in dynamic agroecosystems. Another challenge is that despite extensive research, quantitative comparisons of BCAs’ efficacy across crops, environments, and stress conditions remain limited, reflecting heterogeneity in study designs and response metrics as well as the scarcity of standardized field validation.

Ultimately, sustaining yields and ecological integrity under accelerating climatic and biotic pressures will require adaptive, multifunctional microbial solutions developed through a convergence of microbiome science, plant genetics, ecological modelling, and precision delivery technologies. In this context, I propose a conceptual framework (Figure 1) that advances existing IPM and microbiome-engineering paradigms by integrating (i) microbial design and synthetic consortia, (ii) omics-based screening and functional optimization, (iii) microbiome-informed IPM and precision agriculture, and (iv) host genetics and holobiont optimization across environmental gradients. Through this broad scope, the framework explicitly links near-term, deployable interventions to longer-term innovation pathways, moving beyond descriptive biocontrol strategies toward a systems-level approach for resilient, climate-smart agriculture.
